# Effects of andrographolide on cellular metabolism and mitochondrial function: current evidence and insights into its anticancer mechanisms

**DOI:** 10.3389/fphar.2026.1921579

**Published:** 2026-09-23

**Authors:** Catalina González-Seguel, John Quiroga, Juan Hancke, Pamela Ehrenfeld, Patricio Barrales, Rafael A. Burgos

**Affiliations:** 1 Laboratory of Inflammation Pharmacology and Immunometabolism, Institute of Pharmacology and Morphophysiology, Faculty of Veterinary Sciences, Universidad Austral de Chile, Valdivia, Chile; 2 HP Ingredients Corp., Bradenton, FL, United States; 3 Laboratory of Cellular Pathology, Institute of Anatomy, Histology and Pathology, Faculty of Medicine, Universidad Austral de Chile, Valdivia, Chile

**Keywords:** andrographolide, apoptosis, cancer, ferroptosis, metabolism, mitochondria

## Abstract

Andrographolide, a labdane diterpenoid derived from *Andrographis paniculata*, has demonstrated anticancer activity across multiple preclinical models. Accumulating evidence indicates that andrographolide acts as a multitarget modulator of signaling and metabolic pathways, including PI3K/Akt, MAPK, and AMPK. Reported effects also include context-dependent modulation of Nrf2 signaling, ROS accumulation, loss of mitochondrial membrane potential, alterations in mitochondrial morphology and dynamics, and activation of apoptosis or ferroptosis. However, most studies demonstrate pathway modulation or downstream mitochondrial consequences rather than direct engagement of a validated mitochondrial molecular target. This review integrates current evidence linking cellular metabolism, mitochondrial dysfunction, and regulated cell death to the anticancer effects of andrographolide, while critically examining tumor selectivity, safety, pharmacokinetic limitations, and the predominantly preclinical nature of the available evidence.

## 1 Introduction

Andrographolide is a bioactive compound derived from *Andrographis paniculata*, a plant native to China that has been used for centuries in traditional Asian medicine because of its diverse properties. Among these, its anti-inflammatory, antioxidant, and antiviral effects stand out, which is why its use has been mainly indicated for the treatment of respiratory tract conditions, fever, colds, and the management of various infectious diseases (Lin et al., 2009; Narimanyan et al., 2021).

In recent decades, the study of andrographolide has gained relevance due to its pharmacological potential as an anti-inflammatory agent and possible antineoplastic treatment. Various authors have demonstrated the cytotoxic capacity of andrographolide against a wide variety of tumor cell lines, including breast cancer (Laiso et al., 2024; Li et al., 2022), lung adenocarcinoma (Wang et al., 2022), glioblastoma (Chiu et al., 2019), prostate cancer (Chun et al., 2010), colorectal cancer (Liu et al., 2024), and different types of leukemia (Chen et al., 2017; Liao et al., 2019) (Table 1). Some proposed mechanisms indicate that andrographolide may exert its antitumor action by modulating cellular signaling pathways, reducing metastasis and angiogenesis, deregulating the cell cycle, inducing apoptosis, and synergizing with other antineoplastic drugs (Kumbhar et al., 2024; Li et al., 2020b; Othman and Mohd Azman, 2022; Zhou et al., 2010). However, despite efforts to elucidate its mechanism, the manner in which this compound exerts its properties remains a subject of debate, presenting a critical gap in our understanding necessary for its therapeutic optimization.

**TABLE 1:** Overview of the antitumor activity of andrographolide in human cancer types.

Cancer linage	Mechanism	Cell line	Doses IC_50_ (μM)	References
Cervical cancer	PI3k/akt inhibition and PTEN activation	-HeLa -SiHa	8.14 6.83	Pasha et al. (2024)
Osteosarcoma	PI3k/akt, and Wnt/B-catenin inhibition: reduction of phosphorylation of AKT and GSK3-β	-143B -U2OS -SaoS2 -MG63	4.42 10.97 23.54 16.09	Huang et al. (2022)
Glioblastoma	ERK1/2 regulation Interaction of c-myc with p53	-DBTRG-05MG	42.82	Othman and Mohd Azman (2022)
Nasopharyngeal carcinoma	AMPK Activation: increased expression of LKB1	-C666-1	Not reported	Wu et al. (2018)
Lung cancer	Suppression of aerobic glycolysis: PDK1 inhibition and reduction in lactate generation	-A549 -H292 -H522	102.2 46.17 43.59	Yang E. et al. (2023)
Thyroid cancer	Glycolysis suppression: Lower expression of HK2, PKM2, PFKM, and LDHA proteins	-8505C -CAL62	Not reported	Yang et al. (2021)
Triple-negative breast cancer	Mitochondrial-mediated apoptosis: ROS production, potential membrane loss, caspase activation, increased bax and Apaf-1 and decreased bcl-2 and Bcl-xl expression	-MDA-MB-231 -MCF-7	51.98 61.11	Banerjee et al. (2016)
Lymphoma	Mitochondrial-mediated apoptosis: ROS production, caspase activation, bax conformational change	-Ramos -Granta -HF-1 -SUDHL4	20 40 15 30	Yang et al. (2010)
Chronic myeloid leukemia	Degradation of oncogenic proteins: cleavage in HSP90 and degradation of bcr-abl	-K562	Not reported	Liu et al. (2014)
Hepatocellular carcinoma	Mitochondria-mediated apoptosis: DNA damage, increased ROS and decreased BRCA1, RAD51 and 53BP1	-HepG2 -Huh7 -HCCM	Not reported	Li X.-Y. et al. (2023)
Colon cancer	Mitochondria-mediated apoptosis: Nrf2 inhibition, reduction of OXPHOS, alteration in mitochondrial dynamics	-HT29	9.3	Midde et al. (2025)
Burkitt’s lymphoma	Mitochondria-mediated apoptosis: Interaction with CASP3, CASP8, and JUN. Inhibition of c-Jun	Patient sample: S1 S2 S3	69.98 102.02 135.9	Zeng et al. (2024)
Liver cancer	MPTP and autophagy promotion: disruption of mitochondrial transmembrane potential, increased ROS	-Huh-7	17.6	Chen et al. (2012)
Colorectal cancer	Mitochondrial dysfunction: VDAC interaction and immune activation via cGAS-STING, IFN activation	-CT26	19.38	Wu et al. (2025b)
Multiple myeloma	Ferroptosis: increased ROS, decreased GSH and SOD, increased p-p38, decreased Nrf2, and HO-1	-RPMI8226 -U266	41 39.34	Li W. et al. (2023)
Non-small lung cancer	Ferroptosis and mitochondrial dysfunction: small mitochondria, increased mitochondrial membrane density, and decreased mitochondrial crest	-H460 -H1650	33.16 32.45	Jiaqi et al. (2023)
Acute lymphoblastic leukemia T	Apoptosis and ferroptosis: increase in FACL4, ROS, and morphological alterations in mitochondria	-Jurkat -Molt-4 -Nalm-6	5 11.3 15.9	Doi et al. (2025)
Pancreatic cancer	Ferroptosis: GPX4 Ubiquitination	-BxPC3 -MIA PaCa-2	31.48 46.2	Ullah et al. (2025)
Colorectal cancer	Ferroptosis: dysregulated genes HMOX1, GCLC, and GCLM	-HCT116 -HT29	54.2 28.5	Shimura et al. (2021)
Acute lymphoblastic leukemia	Autophagy inhibition: PI3K/Akt/mTOR pathway activation	-CEM-C1	Not reported	Li et al. (2024)
Histiocytic lymphoma	Autophagy promotion: increased expression of ATG 3, ATG 5, and ATG 7. Increased expression of LC3-II/LC3-I	-U937	8.7	Kumar et al. (2015)
Non-melanoma skin cancer	Autophagy promotion: reduced expression of p62, Beclin1, and LC3II/I proteins PTEN increase	N/A	Not reported	Zhong et al. (2022)

As early as the first half of the 20th century, cellular metabolism was proposed as a distinguishing feature of tumor cells, thanks to the discovery by Otto Warburg, who established that these cells produce energy in the form of ATP through lactic fermentation, even in the presence of oxygen (Warburg effect) (Warburg, 1956). Since then, numerous metabolism-related alterations have been studied, ranging from the overamplification of oncogenic expression factors such as c-myc, which transactivates lactate dehydrogenase A, to the overactivation of the Phosphatidylinositol 3-kinase/Protein Kinase B (PI3K/Akt) pathway, which enables increased glucose uptake, and the higher expression of Hypoxia-Inducible Factor 1-alpha (HIF-1α), which allows for the adaptation of metabolism in oxygen -and nutrient- poor environments (Finley, 2023). The metabolism of tumor cells is not the same in all tissues, and the adaptations depend on the cell type, stage, and location (Shuvalov et al., 2021). However, alterations in nutrient acquisition and processing reflect the cell’s need to survive and proliferate, creating a vulnerability that can be targeted pharmacologically to treat the disease.

Mitochondrial metabolic reprogramming is now well recognized as a driver of tumor aggressiveness, with cancer cells shifting their bioenergetic reliance between oxidative phosphorylation (OXPHOS) and aerobic glycolysis, depending on tumor type and disease stage (Tzeng et al., 2026; Ward and Thompson, 2012). In more aggressive tumors, this shift is often accompanied by mitochondrial dysfunction, including impaired electron transport chain activity, loss of membrane potential, and elevated mitochondrial reactive oxygen species (ROS) production, which paradoxically sustains pro-survival signaling, blunts apoptotic responses, and limits the efficacy of conventional chemotherapy (Sullivan and Chandel, 2014; Wallace, 2012; Wu et al., 2024). Beyond bioenergetics, inter-tumor differences in mitochondrial mass, fusion/fission dynamics, and substrate preference further diversify the responses of cancer cells of distinct lineages to drugs (Vyas et al., 2016; Zong et al., 2016). Against this backdrop, the inconsistent antitumor activity of andrographolide across cancer types may not be coincidental but rather a reflection of the mitochondrial and metabolic context of each tumor.

Several recent reviews have summarized the broad pharmacological and anticancer effects of andrographolide, including its influence on proliferation, migration, signaling pathways, apoptosis, pharmacokinetics, and therapeutic development (Attri et al., 2025; Hu et al., 2024). However, these contributions have generally not examined in a unified and critical manner the strength of the evidence linking metabolic reprogramming, mitochondrial dysfunction, and specific regulated cell-death outcomes. In particular, direct mitochondrial target engagement, tumor-selective mitochondrial effects, adaptive responses, and the translational relevance of the concentrations used in experimental models remain insufficiently resolved. This review addresses these gaps by distinguishing experimentally demonstrated mechanisms from indirect associations and computationally predicted targets.

## 2 Cellular metabolism-related mechanisms

### 2.1 Andrographolide as a multi-target modulator of signaling pathways

Andrographolide consistently demonstrated the ability to interfere with multiple cellular signaling pathways, and these mechanisms have been proposed as key contributors to its antitumor activity (Shaharudin et al., 2024). Importantly, there is a close relationship between the signaling pathways modulated by andrographolide and their roles in essential metabolic processes that maintain cellular homeostasis and survival under both physiological and pathological conditions (Figure 1). For instance, a signaling pathways consistently reported to be modulated by andrographolide is the PI3K/Akt pathway (Tohkayomatee et al., 2022; Xuan et al., 2022; Yadav et al., 2022). The PI3K cascade, including upstream receptor tyrosine kinases, the catalytic subunit PI3K p110α, the downstream kinase Akt, and the negative regulator Phosphatase and tensin homolog (PTEN), is frequently altered in cancer, leading to persistent proliferative signaling that promotes tumor cell survival (Noorolyai et al., 2019).

**FIGURE 1:**
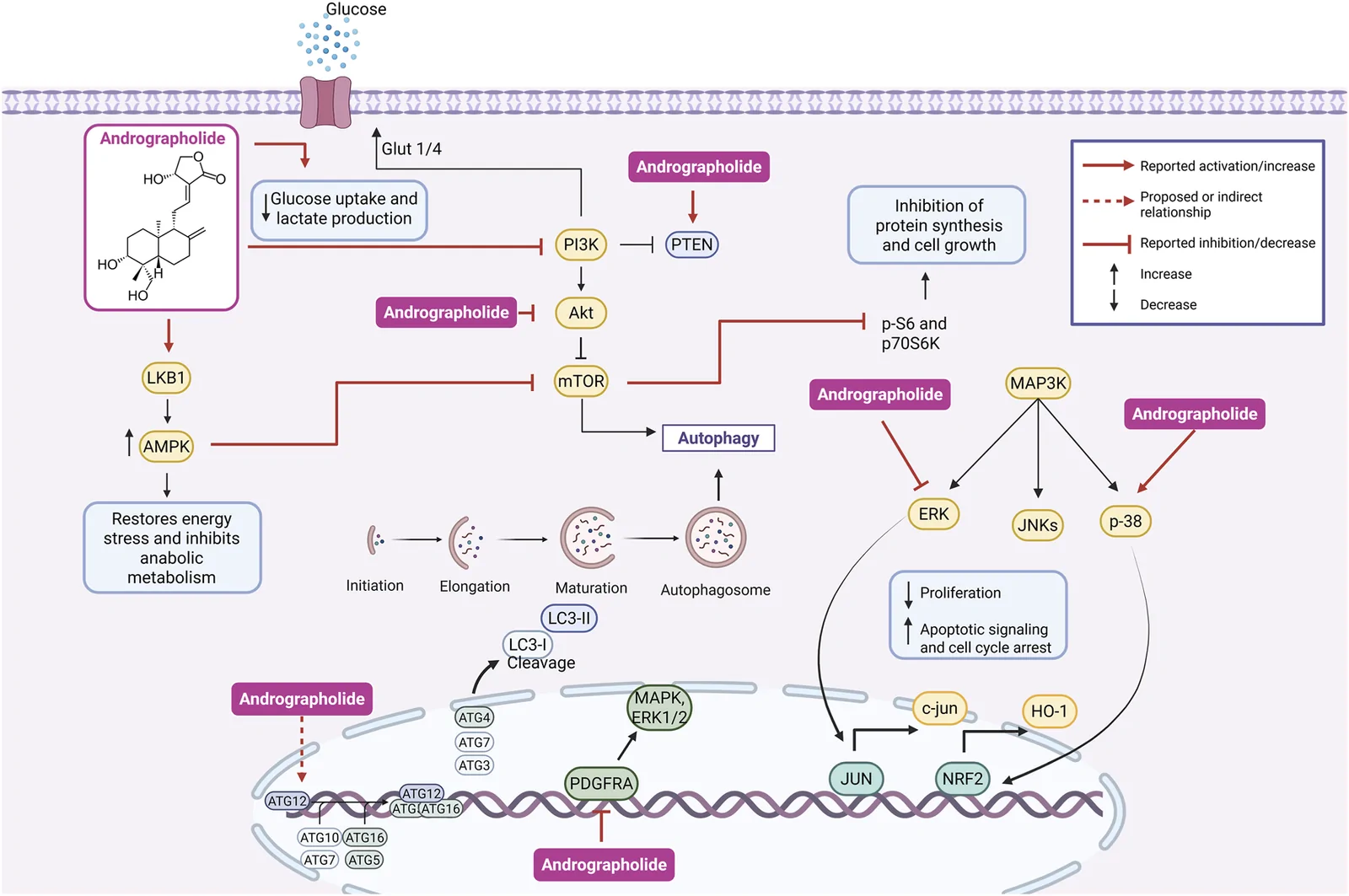
Andrographolide modulates metabolic signaling pathways to promote cell death in tumor cells. Schematic representation of the major signaling pathways targeted by andrographolide in cancer cells, including PI3K/Akt, MAPK, and AMPK cascades. Andrographolide is depicted as the central modulator. The key downstream effects include suppression of proliferative signaling, autophagy activation, cell cycle arrest and apoptosis signaling. Abbreviations: PI3K, phosphatidylinositol 3-kinase; Akt, protein kinase B; MAPK, mitogen-activated protein kinase; AMPK, AMP-activated protein kinase; PTEN, phosphatase and tensin homolog; mTOR, mechanistic target of rapamycin; ERK, Extracellular Signal-Regulated Kinase; JNKs, c-Jun N-terminal Kinases; HO-1, Heme Oxygenase-1; Nrf2, Nuclear Factor Erythroid 2-Related Factor 2; PDGFRA, Platelet-Derived Growth Factor Receptor Alpha; LC3-I/II, Microtubule-Associated Protein 1 Light Chain 3-I/II. Created in https://BioRender.com.

In a cervical cancer study, the authors observed that andrographolide inhibited PI3K/Akt pathway activation while increasing PTEN protein expression in a dose-dependent manner. Furthermore, *in vivo* experiments using mice treated with 15 mg/kg and 30 mg/kg andrographolide showed a reduction in xenograft tumor size. Western blot analyses of proteins involved in the PI3K/Akt cascade also revealed a consistent decrease in pathway-related protein expression (Pasha et al., 2024). Interestingly, these effects were also observed in cervical cancer cell lines resistant to chemotherapeutic agents, such as cisplatin, where andrographolide exerted a synergistic effect that enhanced tumor cell sensitivity to therapy. This was accompanied by marked inhibition of the PI3K/Akt signaling pathway and increased expression of the tumor suppressor protein PTEN (Pasha et al., 2025).

In addition to PI3K/Akt inhibition, it has been proposed that the anti-invasive effects of andrographolide may result from the suppression of both the activity and gene expression of the metalloproteinase MMP-7 (Lee et al., 2010). Notably, this effect was reversed following transfection with an Akt-overexpressing vector in the lung cancer cells. Moreover, using an Electrophoretic Mobility Shift Assay (EMSA), it was demonstrated that reduced AP-1 DNA-binding activity occurred following andrographolide treatment. These findings demonstrate that the reduction in tumor invasiveness may occur through the inactivation of the transcription factors c-Jun and c-Fos, ultimately leading to decreased MMP-7 expression (Lee et al., 2010). Similarly, in osteosarcoma cells 143B, a decrease in Akt phosphorylation at serine 473 and threonine 308 was observed after treatment with andrographolide for 24 h. These results were accompanied by decreased β-catenin levels due to the suppression of GSK3-β phosphorylation at serine 9, which controls β-catenin degradation. In this study, inhibitors of the Wnt/β-catenin pathway (KYA1797K), PI3K/Akt (LY294002), and NF-κB (pyrrolidinedithiocarbamate ammonium, PA) synergistically suppressed osteosarcoma cell proliferation, migration, and invasion (Huang et al., 2022).

It is important to note that, while the downstream consequences of PI3K/Akt inhibition by andrographolide are well-documented across multiple cancer cell lines, further mechanistic studies are required to determine whether PI3K/Akt modulation results from direct target engagement, receptor-level interference, redox-dependent regulation, or indirect effects on upstream pathway components.

The MAPK pathway transduces extracellular and intracellular signals involved in proliferation, differentiation, survival, and migration through successive phosphorylations of MAPK kinase kinase (MAPKKK) and MAPK kinase (MAPKK), culminating in the phosphorylation of one of the main families, such as extracellular signal-regulated kinases (ERK), JNKs, and p38/MAPK, and has been found to be dysregulated in various cancer types (Ullah et al., 2022). Andrographolide has been described as intervening in these pathways by suppressing ERK phosphorylation, thus promoting apoptotic signals in tumor cells by suppressing its activity as an oncogene and inhibiting the transcription of c-Jun and activator protein-1 (AP-1) (Chen et al., 2025; Zhang et al., 2025a). These results were supported by network analysis and molecular docking, which established a relationship between andrographolide and target genes such as MAPK1, MAPK8, MAPK14, MMP9, and Platelet-derived Growth Factor Receptor A (PDGFRA), which encode components of the MAPK and ERK1/2 signaling pathways (Janani et al., 2022; Kumbhar et al., 2024).

Glioblastoma is a highly aggressive brain tumor associated with high mortality rates. In DBTRG-05MG glioblastoma cells, andrographolide induced cell death with an IC_50_ of 13.93 µM after 72 h. In contrast, healthy brain cells (SVGp12) maintained over 50% viability, even at concentrations as high as 200 μM, under the same conditions, highlighting the selective cytotoxicity of andrographolide toward tumor cells (Othman and Mohd Azman, 2022). In contrast to previous, andrographolide treatment increased c-Myc and ERK1/2 expression at the mRNA and protein levels. The authors proposed that ERK1/2 activation enhances c-Myc transcription, which in turn regulates a broad set of target genes, including p53. This signaling cascade may underline the observed G2/M cell cycle arrest and subsequent induction of apoptosis in tumor cells (Othman and Mohd Azman, 2022).

The AMP-activated protein kinase (AMPK) pathway regulates cellular energy homeostasis by sensing changes in AMP/ATP and ADP/ATP ratios and controlling the catabolic and anabolic pathways involved in ATP production through a heterotrimeric kinase composed of three subunits (α, β, and γ), in which the α subunit serves as the catalytic domain that determines the activity of the kinase complex and mediates AMPK regulation of metabolic pathways at both the protein and gene expression levels (Novikova et al., 2015). AMPK is activated under stress conditions when AMP levels increase, as occurs during hypoxia, oxidative stress, ischemia, nutrient deprivation, and persistent muscle contraction (Hardie et al., 2012). In cancer, this pathway is deregulated upstream by the tumor suppressor liver kinase B1 (LKB1), affecting its control and cell cycle arrest functions, favoring glucose utilization (the Warburg effect), and maintaining the stabilization of HIF-1α (Hardie et al., 2012). Although less studied than other cancer pathways, andrographolide can also intervene and promote the activation of the AMPK pathway. Previous studies have shown that in nasopharyngeal carcinoma cells (C666-1), andrographolide increases the expression of LKB1 and decreases the expression of phosphorylated S6 and P70S6, which favors AMPK pathway activity, thus contributing to decreased cell viability (Wu et al., 2018). In addition, in a cellular model of myoblasts (L6), andrographolide promoted the expression of the glucose transporter GLUT4 through the protein kinase C (PKC) pathway and partially through AMPK, facilitating glucose uptake even in insulin-resistant cells, which could be of interest given the connection between the pathophysiology of type 2 diabetes and cancer (Liao et al., 2022).

### 2.2 Metabolic reorganization and glycolysis

Metabolism has been identified as a differential marker of tumor cell behavior, opening up the possibility of identifying vulnerabilities in cancer to halt its proliferation and growth (Hanahan and Weinberg, 2011). In the early 20th century, Otto Warburg observed that tumor cells produce lactate even under oxygen-rich conditions, a phenomenon known as aerobic glycolysis or the Warburg effect (Warburg, 1956). Although this phenomenon was initially interpreted as a reduced reliance on oxidative metabolism, current evidence indicates that glycolysis and mitochondrial respiration function cooperatively to sustain the high proliferative capacity of tumor cells. This metabolic coordination enables the generation of glycolytic intermediates that support biomass synthesis, while limiting excessive electron flux and preserving redox homeostasis (Finley, 2023; Pavlova et al., 2022).

It was reported that, in A549 and H292 lung cancer cells, andrographolide suppresses PDK1 activity, thereby reducing the inhibitory phosphorylation of pyruvate dehydrogenase (PDH) at the E1α subunit. The authors indicate that PDH activity is restored, which favors the conversion of pyruvate into acetyl-CoA instead of lactate. This metabolic shift increases ROS production and promotes the loss of mitochondrial membrane potential. Together, these events contribute to the induction of apoptosis in tumor cells (Yang E. et al., 2023). Conversely, in anaplastic thyroid carcinoma cells (8505C and CAL62), andrographolide suppressed glycolytic activity in a concentration-dependent manner. At higher concentrations (>45 µM), glucose levels in the culture medium remained stable after 48 h, whereas lactate production was maintained at low levels. On the other hand, lower concentrations (<30 µM) were associated with increased glucose consumption and lactate accumulation. Consistently, andrographolide reduced the expression of key glycolytic enzymes, including hexokinase 2 (HK2), pyruvate kinase muscle 2 (PKM2), phosphofructokinase muscle type (PFKM), and lactate dehydrogenase subunit A (LDHA), as shown by Western blotting and PCR analyses (Yang et al., 2021). A similar effect was observed in HCT116 colon cancer cells, where andrographolide enhanced radiosensitivity, partly by suppressing glycolytic activity, as evidenced by reduced glucose consumption, decreased lactate production, and decreased expression of key glycolytic genes, such as PFK1, glucose transporter 1 (GLUT1), and HK2 (Li et al., 2020a).

### 2.3 Autophagy

Autophagy is a conserved cellular degradation pathway that removes damaged organelles, protein aggregates, cytoplasmic components, and intracellular pathogens to maintain homeostasis through the sequestration of this cargo within a double-membrane vesicle, the autophagosome, which subsequently fuses with lysosomes to enable its enzymatic degradation. Selective autophagy is guided by ubiquitination signals and is commonly activated during nutrient deprivation, stress, or cellular remodeling (Li et al., 2020a). Autophagy is regulated by autophagy-related genes (ATGs) that encode proteins involved in cargo recognition, autophagosome formation, vesicle fusion, and lysosomal degradation (Levine and Kroemer, 2019). Various signaling pathways regulate autophagy via stimuli, such as cellular stress and growth factors, that integrate the mTORC1 and AMPK pathways (Bento et al., 2016).

Autophagy can play a dual role in cancer; The literature describes that in early stages it could prevent the continued replication of tumor cells by preventing the accumulation of damaged proteins and organelles. However, in advanced stages, it could favor tumor growth by helping cancer cells survive under stress, thus promoting resistance to treatments and the progression of metastasis (Galluzzi et al., 2015; Vitto et al., 2022).

Andrographolide promotes autophagy and apoptosis in a histiocytic lymphoma cell line (U937) (Kumar et al., 2015). The authors stated that treatment with a strong analog of andrographolide increased the expression of ATG 3, ATG 5, ATG 7, and the ATG 5-ATG 12 conjugate, in a time-dependent manner. Furthermore, key proteins such as LC3-II and LC3-I increased after treatment with andrographolide for 24–48 h, as did LC3 mRNA; these results are strongly supported by electron microscopy visualization of double-membrane autophagosomes that fused with large and small single-membrane vesicles (Kumar et al., 2015). Similar results were obtained in two chondrosarcoma cell lines, SW1353 and Hs 819.T, showing reduced p62 expression and increased Beclin-1 expression and LC3A/B-II/LC3A/B-I ratios following andrographolide treatment. Furthermore, using confocal microscopy the authors demonstrated that andrographolide increased autophagic flux. Cells were infected with an mRFP-GFP-LC3 lentivirus, and andrographolide treatment significantly increased the red-to-green fluorescence ratio, indicating enhanced autophagosome formation and cargo degradation. Notably, these effects were reversed by the autophagy inhibitor chloroquine, suggesting that autophagy contributes to the reduction in cell viability induced by andrographolide in chondrosarcoma cells (Wu et al., 2025a).

Importantly, these findings highlight the need to distinguish changes in autophagy-related proteins from alterations in autophagic flux, as an increased LC3-II/LC3-I ratio and elevated Beclin-1 levels are commonly interpreted as indicators of autophagy induction but do not necessarily reflect increased autophagic flux (Klionsky et al., 2021). In a mouse model of non-melanoma skin cancer induced by long-term UV exposure, the authors reported impaired autophagy, as evidenced by marked increases in p62, phosphorylated p62 (Ser349), Beclin-1, and the LC3-II/LC3-I ratio, despite no significant increase in p62 mRNA levels. Notably, treatment with andrographolide sodium bisulfate reversed these alterations, significantly reducing p62, phosphorylated p62, Beclin-1, and the LC3-II/LC3-I ratio compared with the UV-irradiated model group. These changes were interpreted by the authors as evidence that andrographolide sodium bisulfate restored autophagic flux in UV-induced skin cancer (Zhong et al., 2022).

In contrast, the combined effects of andrographolide and dexamethasone in CEM-C1 acute lymphoblastic leukemia cells enhanced antitumor efficacy by inhibiting autophagy and activating the PI3K/Akt/mTOR pathway (Li et al., 2024). Mechanistically, this effect is associated with lysosomal alkalinization, reduced mRNA and protein expression of key autophagy markers, including Beclin-1 and LC3-II, and increased phosphorylation of PI3K, Akt, and mTOR (Li et al., 2024). In addition, a study in Jurkat cells demonstrated that the decrease in cell viability induced by andrographolide treatment was independent of autophagy. The authors reported no increase in autophagosome formation, as confirmed by the absence of conversion from cytosolic LC3-I to lipidated LC3-II, a specific marker for the formation of this complex (Yang et al., 2016).

Taken together, these findings indicate that the role of autophagy following andrographolide treatment is not uniform but rather depends on tumor type, dose, treatment duration, and lysosomal competence. This context-dependent role of autophagy may explain the contradictory findings in the literature: in some cell lines, andrographolide promotes autophagy that contributes to cell death, whereas in others, autophagy may function as an adaptive response that removes damaged mitochondria and supports tumor-cell survival, such that its inhibition enhances antitumor efficacy. It is important to note that direct evidence linking andrographolide-induced mitochondrial dysfunction to mitophagic flux and treatment resistance remains limited. Experiments combining mitochondrial-damage measurements with PINK1/Parkin-dependent mitophagy assays and pharmacological or genetic autophagy inhibition will be necessary to resolve this relationship.

## 3 Mitochondrial-related mechanisms

### 3.1 Mitochondrial dysfunction

Mitochondria are essential organelles for multiple cellular functions, most notably the maintenance of cellular bioenergetics, regulation of metabolism, and facilitation of processes such as apoptosis, which are crucial for cellular homeostasis and survival (Javadov et al., 2020). In cancer cells, mitochondria are central to bioenergetic adaptation, survival, and evasion of cell death under stressful conditions (Pavlova et al., 2022). Persistent oxidative stress can impair mitochondrial function and activate adaptive responses, including the mitochondrial unfolded protein response, characterized by the increased expression of proteins such as heat shock protein 60 (HSP60) and activating transcription factor 5 (ATF5) (O’Malley et al., 2020). However, the central role of mitochondria in tumor cell survival and metabolic adaptation also makes them attractive to therapeutic targets. Accordingly, mitochondria-targeted anticancer agents, known as “mitocans,” have been developed to selectively disrupt oxidative phosphorylation (OXPHOS), perturb redox homeostasis, and modulate mitochondrial-dependent cell death pathways (Yang Y. et al., 2023).

#### 3.1.1 Mitochondrial membrane potential (Δψm)

The mitochondrial membrane potential (Δψm) is an essential indicator of mitochondrial function and cellular bioenergetic status, generated by the proton gradient established across the inner mitochondrial membrane by the electron transport chain, which stores energy that is subsequently harnessed by ATP synthase for ATP production (Zorova et al., 2018). Sustained disruption of Δψm compromises mitochondrial function, promotes excessive ROS generation and accumulation, and can facilitate the release of pro-apoptotic factors such as cytochrome c into the cytosol. These events amplify cellular stress and damage signals, ultimately contributing to the activation of irreversible cell death pathways (Lebiedzinska-Arciszewska et al., 2025). Given the central role of mitochondrial function in maintaining the high metabolic and biosynthetic demands of cancer cells, disruption of Δψm represents a strategy to selectively compromise tumor cell survival and promote cell death.

In breast cancer cells, andrographolide treatment induced a concentration-dependent decrease in Δψm, a key event associated with mitochondrial dysfunction and subsequent apoptosis, as further supported by changes in apoptotic markers, including a cytochrome c release, caspase activation, reduction in the anti-apoptotic protein Bcl-2 and an increase in the pro-apoptotic protein Bax (Banerjee et al., 2016; Tamang et al., 2022; Suwan et al., 2026). Interestingly, a crude extract of *A. paniculata* reproduced a similar pattern of mitochondrial dysfunction and apoptotic marker modulation in MDA-MB-231 cells, despite being a non-purified formulation standardized only by its andrographolide content. Cytotoxic concentrations were therefore expressed as mg/mL of extract, with IC_50_ values ranging from 0.8 to 7.67 mg/mL. Treatment with the extract induced a marked reduction in Δψm across all three extract formulations containing andrographolide at 10% (1.4 mg/mL), 30% (4.2 mg/mL), and 50% (7.0 mg/mL), with reported reductions of 66.9% ± 0.9%, 73.26% ± 31%, and 69.47% ± 2%, respectively. These changes were accompanied by increased cytochrome c release, decreased Bcl-2 expression, and elevated levels of caspase-3, p53, and p21, supporting the activation of a mitochondria-dependent apoptotic pathway (Laiso et al., 2024). Importantly, these findings cannot be attributed exclusively to andrographolide and should be interpreted as supportive evidence derived from *A. paniculata* extracts rather than from the purified compound.

#### 3.1.2 ROS-dependent mechanisms

Mitochondria are a major source of reactive oxygen species (ROS), particularly through electron leakage from respiratory chain complexes I and III. Under conditions of redox imbalance, excessive ROS accumulation can overwhelm cellular antioxidant defenses, causing oxidative damage to proteins, lipids, and DNA and ultimately promoting the activation of apoptotic cell death pathways (Valko et al., 2006). Free radicals are highly reactive molecules containing unpaired electrons and can be broadly classified into two main categories: radical species, such as hydroxyl radicals (•OH) and superoxide anions (O_2_•^−^), which contain unpaired electrons, and non-radical reactive species, such as hydrogen peroxide (H_2_O_2_) (Ju et al., 2024).

ROS act as secondary messengers in cell signaling and are involved in a wide range of biological processes in both healthy and malignant cells; however, their effects are highly dependent on their concentration, allowing ROS to play a dual role in regulating tumor cell survival and death (Harris and DeNicola, 2020). At low or moderate concentrations, ROS serve as signaling transmitters that stimulate angiogenesis, invasion, migration, and cell proliferation, thereby promoting cancer survival and spread. In contrast, at high concentrations, ROS can activate signaling pathways that promote apoptosis in cancer cells, as the oxidation of DNA bases and strand breaks affects their ability to replicate and/or repair themselves, in addition to altering cellular components, such as chromosomes, proteins, and phospholipids, leading to cell cycle arrest and ultimately, cell death (Chasara et al., 2023).

Andrographolide has been used for centuries as a natural compound with potential antioxidant properties. Part of this mechanism is due to its ability to regulate the nuclear factor erythroid 2–related factor 2 (Nrf2) signaling pathway, which allows it to neutralize ROS and indirectly contribute to the protection of mitochondrial integrity by inhibiting pro-oxidant enzymes and/or activating antioxidant enzymes (Mussard et al., 2019). However, the literature is controversial regarding whether andrographolide increases or decreases free radical concentrations, which may vary depending on the dose and cell type in which it is used. Studies on various tumor cell lines have shown that at high concentrations of andrographolide, ROS levels can be consistently elevated (Liu et al., 2014; Liu et al., 2022; Mongkon et al., 2025; Wang et al., 2024). Nevertheless, ROS accumulation induced by high-dose andrographolide varies by cell type and treatment duration. In Ramos, Granta-519, HF-1, and SUDHL4 lymphoma cell lines, andrographolide significantly increased ROS levels after 1.5–48 h of exposure to concentrations above 10 µM. (Yang et al., 2010). In contrast, in H292 and A549 non-small cell lung cancer cells, the use of andrographolide for 6 h induced this increase at concentrations between 20–75 μM and 20–50 μM, respectively (Yang E. et al., 2023). It has been proposed that andrographolide-induced ROS accumulation promotes the cleavage of heat shock protein 90 (HSP90), thereby impairing its chaperone function. This facilitates ubiquitin–proteasome-mediated degradation of oncogenic proteins, such as Bcr-Abl, and prevents the maturation and stabilization of v-Src, both of which contribute to cancer cell survival (Liu et al., 2014).

It has been proposed that the increase in ROS production by andrographolide is closely related to DNA damage, as reflected by chromatin condensation, DNA fragmentation, and the presence of irregular edges around the nucleus, leading to tumor cell death (Roy et al., 2025). In hepatocellular carcinoma cells, an increase in γ-H2AX (H2AX phosphorylated at ser139) was observed, accompanied by a decrease in key DNA damage response proteins such as BRCA1, RAD51, and 53BP1, demonstrating that andrographolide promotes their degradation through the proteasome, which appears to be regulated by p62 (Li X.-Y. et al., 2023). In addition to the mechanisms described above, andrographolide may exert antineoplastic effects by inhibiting Nrf2, a key transcription factor activated in response to oxidative stress that promotes the expression of genes involved in detoxification, ROS clearance, and cytoprotection (Guerrero-Hue et al., 2020). Although Nrf2 activation plays an important role in maintaining cellular redox homeostasis, its persistent and aberrant activation in cancer cells can enhance their antioxidant capacity, facilitate the removal of excess intracellular ROS, reduce oxidative DNA damage, and protect tumor cells from oxidative stress-induced apoptosis (Jiao et al., 2026). In colon cancer spheroids, the combination of andrographolide and melatonin exerted a synergistic antitumor effect and reduced Nrf2 gene expression, suggesting that this combination may compromise the antioxidant defense of tumor cells, increase oxidative stress, and enhance their susceptibility to cell death (Midde et al., 2025).

#### 3.1.3 Mitochondrial dynamics

When mitochondrial damage exceeds the capacity of repair mechanisms, mitochondrial quality control pathways are activated, including changes in mitochondrial dynamics through the coordinated processes of fusion and fission, which are essential for maintaining mitochondrial and cellular homeostasis (Youle and van der Bliek, 2012). Fusion, mediated by mitofusins (MFN1 and MFN2) on the outer mitochondrial membrane and optic atrophy 1 (OPA1) on the inner membrane, promotes the formation of interconnected mitochondrial networks, facilitating the exchange of mitochondrial contents and helping to maintain mitochondrial integrity. In contrast, fission is primarily mediated by the GTPase dynamin-related protein 1 (DRP1) and its recruitment factors, including mitochondrial fission factor (MFF), mitochondrial fission protein 1 (FIS1), and mitochondrial dynamics proteins of 49 and 51 kDa (MiD49 and MiD51) (Creus et al., 2026). In cancer cells, mitochondrial dynamics are frequently altered by changes in gene expression, genetic and epigenetic regulation, and post-translational modifications of proteins involved in mitochondrial remodeling, disrupting the balance between mitochondrial fission and fusion and contributing to the metabolic adaptation and cellular plasticity that support tumor growth and progression (Rossi et al., 2026).

Andrographolide alters the expression of key proteins involved in mitochondrial dynamics when combined with melatonin, as evidenced by decreased levels of phosphorylated DRP1, MFN1, and OPA1 (Midde et al., 2025), suggesting disruption of the coordinated balance between mitochondrial fission and fusion. This is consistent with others reports describing the ability of andrographolide to modulate mitochondrial fission (Geng et al., 2019; Lu et al., 2026; Suzuki et al., 2026). Mitochondrial dynamics is a determinant of mitochondrial morphology and function (Tábara et al., 2025). In this context, ultrastructural analysis of a non-small cell lung cancer cell line revealed that andrographolide treatment resulted in smaller mitochondria, increased membrane density, and altered cristae organization, indicating substantial remodeling of mitochondrial architecture (Jiaqi et al., 2023). Furthermore, andrographolide treatment reduced TOM20 (Suwan et al., 2026), OXPHOS complex proteins, and MRPS6 levels (Midde et al., 2025), suggesting alterations in mitochondrial content, protein homeostasis, and mitochondrial protein synthesis, with potential consequences for oxidative phosphorylation and ATP production. Given the critical role of mitochondrial dynamics and cristae organization in maintaining mitochondrial function, these structural and molecular alterations may contribute to andrographolide-induced mitochondrial bioenergetic dysfunction.

### 3.2 Apoptosis

Apoptosis is a programmed form of cell death through which organisms eliminate cells that are no longer required or cannot be repaired due to extensive DNA damage (Tang et al., 2012). This process can be initiated through two interconnected pathways, the extrinsic and intrinsic pathways, which ultimately converge on the activation of mitochondrial apoptotic signaling in response to stimuli such as ROS accumulation, physical injury, metabolic dysfunction, or developmental and physiological signals that commit the cell to death (Redza-Dutordoir and Averill-Bates, 2016). The extrinsic pathway is activated by death receptors such as FAS or TNFR-family members, which recruit adaptor proteins and activate caspase-8. Caspase-8 can directly activate effector caspases or, in cells requiring mitochondrial amplification, cleave BID to truncated BID (tBID), thereby promoting BAX/BAK-dependent mitochondrial outer-membrane permeabilization (MOMP). In the intrinsic pathway, intracellular stress activates pro-apoptotic BCL-2-family proteins, leading to MOMP and the release of cytochrome c. Cytosolic cytochrome c binds APAF1 and procaspase-9 to assemble the apoptosome, which activates caspase-9 and subsequently caspases-3 and -7 (Bock and Tait, 2020; Dadsena et al., 2021; Mustafa et al., 2024). In cancer, both the extrinsic and intrinsic apoptosis pathways can be disrupted through the overexpression, reduced expression, or inactivation of key apoptotic proteins, as well as through genetic alterations affecting genes involved in the initiation and execution of programmed cell death, including the overexpression of anti-apoptotic proteins such as BCL-2, deletions in FASR/TNFRSF6 that disrupt apoptotic signaling, mutations in procaspases-9, -3, and -7 that impair the apoptotic cascade, and deletions of pro-apoptotic proteins such as BIM and BAX (Kulbay et al., 2022).

Several studies have demonstrated that andrographolide can increase caspase levels and promote apoptosis signaling; For example, a study in monocytic leukemia cells (U937) reported that the activity of caspase-3, -8, and -9 increased by 8, 3, and 5 times, respectively, compared to the control when treated for 48 h with a 5.4 μM (IC_50_) analog of andrographolide (Kumar et al., 2015). Furthermore, the authors, upon using a strong inhibitor of caspase activity (Z-VAD-fmk), found that they needed to increase the dose from 5.4 μM to 27 μM to achieve the IC_50_, confirming the crucial role of caspases (Kumar et al., 2015), similar to what occurs in lung, leukemia, multiple myeloma, and acute myeloid leukemia cell lines (Doi et al., 2021; Hodroj et al., 2018; Yang E. et al., 2023). In cells from patients with follicular lymphoma and diffuse large B-cell lymphoma, andrographolide has demonstrated an antiproliferative effect, which is related to the increase and accumulation of apoptotic proteins such as bax and caspase 3, as well as an increase in poly(ADP-ribose) polymerase (PARP), a protein involved in DNA repair that is typically elevated in apoptotic processes as a result of a regulatory mechanism (Yang et al., 2010).

Likewise, in MDA-MB231 cells, andrographolide increased the expression of the pro-apoptotic protein bax and decreased the expression of the anti-apoptotic protein Bcl-2, in addition to triggering the cleavage of PARP protein, thus demonstrating that andrographolide is strongly involved in activating the apoptotic cascade (Tamang et al., 2022). These results are supported by an *in silico* analysis that demonstrated the interaction of andrographolide with Bcl-xL protein, identifying it as a potential therapeutic target for the treatment of various types of cancer (Thai et al., 2025). In Burkitt lymphoma, molecular docking analyses have identified CASP3, CASP8, and JUN as potential andrographolide-associated targets. The authors proposed that increased caspase-related gene activity may suppress JUN transcription, thereby reducing the expression of the oncogenic transcription factor c-Jun (Zeng et al., 2024). Interestingly, other derivatives of andrographolide have shown similar effects. A novel pyridazinone-based andrographolide derivative induced a high rate of cell death in gastric cancer cells at relatively low concentrations, with IC_50_ values of 3.92 and 2.44 μM after 48 and 72 h of treatment respectively (Zhang et al., 2025b). This effect was accompanied by the dose-dependent downregulation of Bcl-2 and upregulation of Bax, cleaved caspase-3, and cleaved PARP, supporting the involvement of mitochondrial-mediated apoptotic processes in the antitumor effects of andrographolide derivatives.

Opening of the MPTP is a critical event in the induction of tumor cell death. This process is triggered by factors such as calcium overload, oxidative stress, and mitochondrial depolarization, which cause mitochondrial dysfunction and the subsequent release of pro-apoptotic factors, such as cytochrome c, a key signal in activating this process (Bock and Tait, 2020; Waseem and Wang, 2023). In a study on human liver cancer cells, treatment with andrographolide induced cell death through autophagy by altering the ΔΨm and increasing ROS levels, both of which are correlated with the function of the MPTP. The authors inhibited a component of the MPTP, cyclophilin D, by using cyclosporine A and siRNA, which resulted in significant suppression of andrographolide-induced cytotoxicity, showing that cyclophilin D plays a key role in mediating andrographolide-induced cytotoxicity in liver cancer cells (Chen et al., 2012).

Another proposed mechanism of action for andrographolide is the activation of the cGAS-STING pathway through direct mitochondrial damage and interaction with the voltage-dependent anion channel (VDAC) protein (Wu et al., 2025b). VDAC is a protein located in the outer mitochondrial membrane that can oligomerize or form pores, allowing the release of mitochondrial content into the cytosol, including mitochondrial DNA (mtDNA), thereby contributing to mitochondrial damage recognition signals and activating innate immune mechanisms (Picca et al., 2021). In colorectal cancer cells, andrographolide treatment reduced ΔΨm, increased mitochondrial ROS levels, opened the MPTP, and significantly increased the release of oxidized mitochondrial DNA (ox-mtDNA) into the cytosol. When the latter is released, DNA sensors such as cGAS are activated, producing the second messenger cGAMP, which activates transcription factors such as IRF3 and NF-κB upon binding to the STING protein, leading to the expression of type I interferon (IFN) and ultimately the activation of innate immunity. Treatment with andrographolide increased the expression of cGAS, STING, and p-IRF3 proteins, as well as IFN-β levels in a dose-dependent manner, supporting a mechanism linking mitochondrial function to andrographolide’s ability to promote antitumor immune responses (Wu et al., 2025b). Interestingly, the interaction between VDAC and hexokinase 2 (HK2), the rate-limiting enzyme of glycolysis, has been shown to protect against cell death by preventing the release of cytochrome c through VDAC (Arzoine et al., 2009). This mechanism has prompted the investigation of HK2 inhibitors aimed at disrupting this interaction, which, in the context of cancer cells, may promote apoptosis and enhance tumor control (Zheng et al., 2021). In a molecular docking and simulation study, andrographolide was evaluated for its binding affinity to HK2. In this computational model, andrographolide exhibited a more favorable docking score than glucose-6-phosphate and formed a stable predicted complex with HK2 during molecular-dynamics simulation (Swargiary and Mani, 2021). These findings identify HK2 as a candidate interaction partner; however, direct biochemical binding, enzyme inhibition, target engagement, and functional rescue have not yet been demonstrated.

Given the central role of mitochondria in cell viability and death, recent studies have explored the mitochondria-targeted delivery of andrographolide as an anticancer strategy. Using a triphenylphosphonium, polyethylene glycol, and polycaprolactone nanoparticle system (TPP-PEG-PCL) in 4T1 breast cancer cells, researchers showed that andrographolide accumulated in mitochondria, markedly inhibited cell proliferation and migration, increased ROS production, and induced the loss of ΔΨm (Yuan et al., 2025). Nevertheless, a primary mitochondrial molecular target of unmodified andrographolide has not been conclusively validated. Moreover, mitochondrial accumulation demonstrated using TPP-conjugated nanoparticles reflects the targeting properties of the delivery system and should not be extrapolated to free andrographolide.Collectively, available evidence points to several candidate mechanistic nodes, including HK2- VDAC-associated signaling, cyclophilin D-dependent MPTP regulation, VDAC-mediated mitochondrial stress linked to activation of the cGAS–STING–IFN-β axis and mitochondrial protein-homeostasis pathways. Functionally, we propose that andrographolide-induced mitochondrial dysfunction, characterized by loss of ΔΨm and ROS accumulation, may trigger MPTP opening, leading to cytochrome c release and subsequent apoptosome formation, ultimately contributing to apoptosis (Figure 2).

**FIGURE 2:**
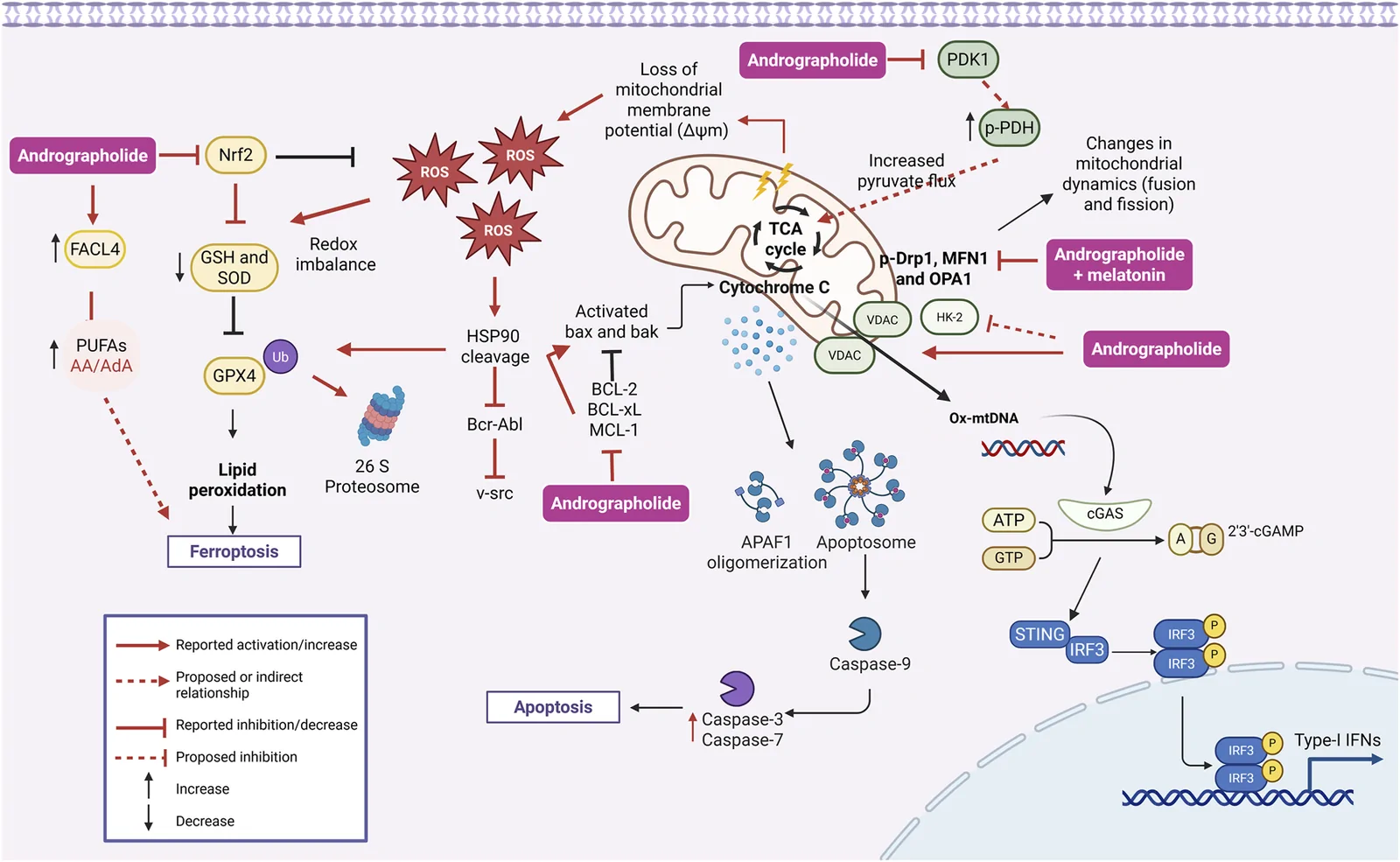
Andrographolide alters cellular redox balance and promotes mitochondrial dysfunction. Proposed model of andrographolide-induced mitochondrial disruption in cancer cells. Andrographolide inhibits Nrf2-mediated antioxidant signaling and mitochondrial damage, leading to a loss of mitochondrial membrane potential (Δψm) and increased intracellular ROS production, promoting the release of cytochrome c and activation of the intrinsic apoptosis pathway. VDAC-mediated mitochondrial stress linked to activation of the cGAS–STING–IFN-β axis is represented, together with concurrent effects on mitochondrial dynamics, reflected by altered abundance of the dynamics-related proteins DRP1, MFN1, and OPA1, and on OXPHOS complex activity. Cell death outcomes include apoptosis and ferroptosis. Abbreviations: ROS, reactive oxygen species; Nrf2, nuclear factor erythroid 2-related factor 2; KEAP1, kelch-like ECH-associated protein 1; DRP1, dynamin-related protein 1; MFN1, mitofusin 1; OPA1, optic atrophy protein 1; OXPHOS, oxidative phosphorylation; GPX4, glutathione peroxidase; FACL4, Fatty acid-CoA ligase 4; GSH, Reduced Glutathione; SOD, Superoxide Dismutase; VDAC, Voltage-Dependent Anion Channel; PDK1, Pyruvate Dehydrogenase Kinase 1; PDH, Pyruvate Dehydrogenase; Ox-mtDNA, Oxidized Mitochondrial DNA; cGAS, Cyclic GMP-AMP Synthase; STING, Stimulator of Interferon Genes; IRF, Interferon Regulatory Factor; Type-I IFNs, Type I Interferon; HSP90, Heat Shock Protein 90. Created in https://BioRender.com.

### 3.3 Ferroptosis

Ferroptosis is an alternative pathway of cell death, driven by iron-dependent lipid peroxidation and fundamentally linked to cellular metabolic state and redox balance, with the mitochondria serving as a central hub for both its execution and regulation (Jiang et al., 2025). Mitochondria contribute to ferroptotic signaling through the generation of reactive oxygen species (ROS) via the electron transport chain, the modulation of the tricarboxylic acid (TCA) cycle, and the management of iron and calcium homeostasis (Sun et al., 2023). Specialized mitochondrial defense systems, such as DHODH and mitochondrial GPX4, act as critical barriers against lethal lipid peroxidation, determining the cellular threshold for death (Karło et al., 2026). In certain types of tumor cells, such as mesenchymal or undifferentiated cells, ferroptosis may be more effective than apoptosis, making it a potential target for cancer treatment, especially for refractory cancers (Zhou et al., 2024; Zhang et al., 2026).

Andrographolide has been proposed to induce ferroptosis in different cancer models; In multiple myeloma cells, treatment with 20–40 µM andrographolide induced several ferroptosis-related changes, including increased ROS production, reduced GSH and SOD levels, increased p38 phosphorylation, and decreased Nrf2 and HO-1 expression. Notably, pharmacological inhibition of p38 with SB202190 partially rescued cell viability, reduced p38 phosphorylation, restored Nrf2, HO-1, and GPX4 levels, and decreased Fe^2+^ accumulation and lipid peroxidation. These findings support the involvement of the p38/MAPK–Nrf2/HO-1 axis in andrographolide-induced ferroptosis in multiple myeloma cells (Li W. et al., 2023). Consistent with these findings, andrographolide also promoted ferroptosis in non-small cell lung cancer cell lines (H460 and H1650), as evidenced by increased intracellular ROS, malondialdehyde (MDA), lipid peroxidation, and free iron levels, together with GSH depletion and downregulation of the ferroptosis-suppressing proteins GPX4 and SLC7A11 (Jiaqi et al., 2023). In the same study, andrographolide suppressed tumor growth and lung metastasis in Lewis lung carcinoma-xenografted C57BL/6 mice and reduced GPX4 and SLC7A11 expression. Importantly, these antitumor effects were attenuated by co-treatment with the ferroptosis inhibitor ferrostatin-1, further supporting a role for ferroptosis in the antitumor activity of andrographolide (Jiaqi et al., 2023).

It is possible that andrographolide activates more than one cell death pathway, as occurs in T-cell acute lymphoblastic leukemia cells (Jurkat and Molt-4), where markers of both apoptosis and ferroptosis are observed (Doi et al., 2025). Andrographolide increased the expression of Fatty acid-CoA ligase 4 (FACL4, also known as ACSL4; Acyl-CoA Synthetase Long-Chain Family Member 4), a protein responsible for the biosynthesis and esterification of polyunsaturated fatty acids (PUFA), a process that promotes lipid peroxidation and the subsequent ferroptosis pathway (Zhou et al., 2024). Furthermore, increased FACL4 expression is accompanied by a significant increase in ROS, loss of Δψm, and morphological changes in mitochondria, revealing alterations in mitochondrial activity (Doi et al., 2025). Glutathione peroxidase 4 (GPX4) is an antioxidant enzyme that protects against lipid peroxidation and is considered a negative regulator of ferroptosis. In pancreatic cancer, andrographolide binds to the chaperone protein HSP90, targeting GPX4 for ubiquitination and promoting its elimination (Ullah et al., 2025). Furthermore, andrographolide treatment has been shown to alters the expression of key genes involved in the induction of ferroptosis *in vitro* and *in vivo* (Shimura et al., 2021). In colorectal cancer cells (HCT116 and HT29), as well as in a murine xenograft model through RNA extraction from xenografted tumors, researchers observed increased expression of genes such as HMOX1, GCLC, and GCLM, which are involved in iron metabolism and glutathione (GSH) metabolism, two critical processes in ferroptosis regulation. Collectively, these findings support the role of andrographolide in promoting ferroptosis, a process closely linked to cellular metabolism and redox homeostasis, highlighting its potential as a promising therapeutic strategy for certain cancers that exhibit resistance to classical cell death pathways, such as apoptosis (Shimura et al., 2021).

## 4 Limitations

Despite the promising antitumor effects of andrographolide, several challenges still limit its translation from preclinical studies to clinical applications. These limitations are primarily related to its safety profile, particularly at higher doses, and its unfavorable physicochemical properties, which contribute to suboptimal pharmacokinetics. Andrographolide exhibits very poor aqueous solubility and is generally described as sparingly soluble in water, with a reported lipophilicity value of XLOGP3 = 2.16 (Song et al., 2022). Furthermore, its oral bioavailability is exceptionally low, which has been attributed to extensive biotransformation and P-glycoprotein-mediated efflux (Ye et al., 2011). Regarding its safety profile, andrographolide has been associated with mild to moderate adverse effects, including gastrointestinal discomfort, dizziness, itching, and dysgeusia (Shang et al., 2022; Songvut et al., 2023). However, more severe adverse events, such as anaphylaxis and acute kidney injury have also been reported (Shang et al., 2022; Zhang et al., 2014) while some animal studies have indicated potential effects on fertility parameters (Ogundola et al., 2021). In a preclinical model, andrographolide sodium bisulfite induced dose-dependent renal histopathological changes, including tubular dilation and congestion, accompanied by alterations in the renal mitochondrial proteome (Xing et al., 2015). However, these nephrotoxic effects -associated with mitochondrial mechanisms- have primarily been reported in animal studies using relatively high or repeated doses, and current clinical evidence does not indicate that andrographolide or its derivatives consistently cause severe or irreversible renal damage. Further dose-response and reversibility studies are therefore needed to better define the safety margins of andrographolide-based therapies.

Finally, evidence supporting tumor selectivity remains limited and heterogeneous. Although some studies report greater cytotoxicity in malignant cells than in non-malignant comparator cells (Banerjee et al., 2016; Laiso et al., 2024; Othman and Mohd Azman, 2022), these comparisons frequently involve cell lines of different origin, transformation status, or proliferative capacity. Moreover, reduced viability related to mitochondrial disfunction in tumor cells does not demonstrate preferential accumulation or selective action within tumor-cell mitochondria. Except for engineered mitochondria-targeted delivery systems, the available evidence does not establish that free andrographolide preferentially accumulates in tumor mitochondria over mitochondria from non-tumor cells, leaving a framework of additional doubts about potential adverse effects on healthy cells that have not yet been identified.

## 5 Conclusion

Tumor cells rely on functional mitochondria and metabolic pathways to sustain the continuous production of amino acids, lipids, and nucleotides required for their proliferation and survival. Under stress conditions that would otherwise trigger cell death, cancer cells can activate protective mitochondrial responses to preserve bioenergetic and metabolic homeostasis. Therefore, therapeutic strategies targeting mitochondrial vulnerabilities remain promising for cancer treatment. In this context, andrographolide represents a potential multitarget antitumor agent, as it modulates multiple signaling, metabolic, and cell death pathways, thereby limiting tumor cells’ ability to adapt and survive in adverse conditions. Overall, the available evidence supports mitochondria as an important convergence node for the metabolic, redox, and regulated cell-death effects associated with andrographolide. However, free andrographolide cannot yet be considered a validated mitochondria-targeted anticancer agent because preferential mitochondrial accumulation and direct engagement of a mitochondrial molecular target have not been conclusively demonstrated. Further studies should integrate direct measurements of respiration and ATP synthesis, mechanism-specific rescue experiments, mitophagic flux, tissue-matched tumor selectivity, pharmacokinetics, and safety before these preclinical findings can be translated into therapeutic applications. This review highlights the mechanistic evidence supporting the use of andrographolide as a promising candidate for anticancer drug development. Nevertheless, further preclinical and clinical studies are required to validate and translate these *in vitro* findings into therapeutic applications.
